# Predictors of Gaming Disorder or Protective from It, in a French Sample: A Symptomatic Approach to Self-Regulation and Pursued Rewards, Providing Insights for Clinical Practice

**DOI:** 10.3390/ijerph19159476

**Published:** 2022-08-02

**Authors:** Sophia Achab, Stephane Rothen, Julie Giustiniani, Magali Nicolier, Elizabeth Franc, Daniele Zullino, Frederic Mauny, Emmanuel Haffen

**Affiliations:** 1 Department of Psychiatry, Faculty of Medicine, Geneva University, 1205 Geneva, Switzerland; elizabeth.megan.franc@gmail.com (E.F.); daniele.zullino@hcuge.ch (D.Z.); 2 Research Centre for Statistics, Faculty of Economy and Management, Geneva University, 1205 Geneva, Switzerland; stephane.rothen@hcuge.ch; 3 Centre d’Investigation Clinique, INSERM CIC 1431, Centre Hospitalier Universitaire de Besançon, 25000 Besançon, France; giustiniani.julie@gmail.com (J.G.); mnicolier@chu-besancon.fr (M.N.); emmanuel.haffen@univ-fcomte.fr (E.H.); 4 Laboratoire de Recherches Intégratives en Neurosciences et Psychologie Cognitive (UR LINC), Université Bourgogne Franche-Comté, 25000 Besançon, France; 5 Unité de Méthodologie en Recherche Clinique, Épidémiologie et Santé Publique, CIC Inserm 143, University Hospital, 25000 Besançon, France; frederic.mauny@univ-fcomte.fr; 6 UMR CNRS 6249 Chrono Environnement, Franche-Comté University, 25000 Besançon, France; 7 Fondation FondaMental, 94000 Créteil, France

**Keywords:** gaming disorder, MMORPG, motivations, benefits, sensation seeking, control, impulsivity, BIS-10

## Abstract

Gaming disorder (GD) is a new health condition still requiring a lot of evidence established around its underlying and related psychological mechanisms. In our study we focused on Massively Multiplayer Online Role Playing Games (MMORPGs), a specific very popular and engaging game genre, to determine that benefit, motivation and control aspects could be predictive of a dysfunctional engagement in gaming. In total, 313 participants were recruited from private forums of gamers between May 2009 and March 2010. They filled out a questionnaire on their socio-demographic data and their weekly gaming time. They also completed different psychometric assessments such as the DSM IV-TR criteria for substance dependence adapted to gaming such as the Dependence Adapted Scale (DAS), the external rewards they expected from gaming (External Motives), the expected internal reward they expected from gaming (Internal Motives), the Zuckerman Sensation Seeking Scale (ZSSS), and the Barratt impulsiveness Scale (BIS-10). Results showed that some psychological factors related to online gaming represented risk factors for GD in participants (i.e., competition and advancement motives, reduced anxiety, solace, greater personal satisfaction, and sense of power), whereas some others were found to be protective factors from GD (i.e., recreation, enjoyment and experience seeking) in participants. Additionally, the study found that disinhibition, boredom susceptibility, thrill and adventure seeking, and high impulsivity were correlated to GD in participants. In conclusion, not only motives for gaming and impulsivity could be predictors for GD, but maladaptive coping strategies based on experienced relief in-game from negative feelings (anxiety and boredom) or experienced improvement in-game of self-perception (personal satisfaction, sense of power) could play as well a role of negative reinforcers for GD. Some benefits from gaming, typically entertainment and enjoyment, are shown to be protective factors from GD, playing the role of positive reinforcing factors. They are worthy of being identified and promoted as functional gaming habits. These findings can feed the clinical and health promotion fields, with a more in-depth understanding of diverse psychological factors in gamers, identifying those at risk for GD and those protective from it. The current work can foster a more balanced approach towards gaming activities, taking their opportunities for mankind and controlling for their adverse effects in some individuals.

## 1. Introduction

### 1.1. Gaming Disorder Is an Addictive Disorder

The video game, which is the best-selling cultural product in the world, owes its extension in part to the development of the internet and to the increasing ubiquity of digital technologies accessible through different mediums; computers, consoles, and smartphones. The multiplication of game interfaces and the increasing of the time spent on the Internet have in some cases led to the emergence of a new disorder, gaming disorder (GD) [1]. Thus, if for most of consumers this activity remains recreational, for another proportion of them, this activity can present addictive patterns, leading to many negative consequences for gamers’ lives [2,3]. GD has recently been recognized as a health condition by new international classification of diseases systems. More precisely, it appears in Section 3 for the non-substance addiction category of the DSM-5 as a condition warranting further research under the term “Internet Gaming Disorder” [4]. It was included in 2018 in “Addictive disorders and addictive behaviors”, a new diagnostic category of the 11th International Classification of Diseases (ICD-11) released in February 2022 by the World Health Organization (WHO) under the term “Gaming Disorder” (GD) [1,5]. The acknowledgement of GD as an addictive disorder had started in front of the several common symptoms with substance use disorders (SUDs), as well as the observation of similar neural and biological mechanisms [6,7,8,9,10,11,12,13]. Thus, as with other addictive behaviors, there are many reasons for the transition from controlled behavior to addiction. Prevalence rates vary among populations, gender and age: in Europe and North America, they usually range between 0–10%, while higher rates are found in Asian countries with ranges between 10–15% [1], and higher over males and youth.

### 1.2. Massively Multiplayer Online Role-Playing Games (MMORPGs)

GD concerns many video game genres, and consequently many personality profiles and reasons to play [14]. But one game genre, the MMORPG, is particularly popular and more likely to induce addictive patterns of use [14].

The main attractors of MMORPGs seem to be (a) the permanent evolving virtual world (even if the player is not connected to the game), (b) the social aspect, continuously linking thousands of gamers (through their graphical representation in-game, known as an avatar), who can group (i.e., in guilds) and communicate to achieve the same objectives in a duplicated version (server) of the virtual world, and (c) the reinforcing property of the game (intermittent and constant rewards delivered through the gamers’ progression) [15]. In addition to the loss of control which is common to all addictions, individuals playing MMORPGs can suffer from GD and experience negative consequences on their social (e.g., isolation, neglected activities and interpersonal conflicts) and professional life (e.g., cyber slacking or reduced productivity) [16]. They can also suffer from health-related issues (e.g., sleep disorders, sedentary lifestyle), and psychological disturbances (e.g., negative mood, irritability, confusion between the virtual and the real self) [2,16,17,18]. With respect to the wide range of game genres, while GD appears to be a stable diagnosis, it is recommended that the game genre should be considered and assessed to understand the rewarding components which can trigger addictive patterns of use; motives to play changing from a game genre to another [14]. In view of these different aspects of the popularity and the risk of addiction linked to MMORPGs, the focus on this genre and on the most popular game ever, World of Warcraft (WoW), seems particularly relevant to study engagement and GD, even after more than a decade after its release.

### 1.3. Motives to Play MMORPGs

Individual motives mediate important determinants of some behaviors, such as alcohol use [16]. Examining motives to game online could have the same relevance in understanding the propensity of some individuals to excessively game. Motivations for playing MMORPGs have been theorized, examined and can be thought of as the expected positive reward associated with the gaming experience. We can mention the motivation questionnaire developed by Yee based on three categories and 10 dimensions inspired by Bartle’s model [17]: *achievement* (advancement, mechanics, competition), *social* (socializing, relationship, teamwork) and *immersion* (discovery, role playing, customization, escapism) [18]. Yee’s model has been designed specifically for the MMORPG, whereas Demetrovics & al. [19] who do not focus on one game genre, identified them as motives to play: *social*, *competition*, *omnipotence-power*, *skill development*, *coping-escape*, *recreation* and *fantasy*. Each category and dimension represent a possible way of perceiving the expected reward while gaming, in an external representation (i.e., rewards related to the gaming environment, the gaming character and the gameplay) or in an internal representation such as the experienced psychological benefits in relation to the play activity (i.e., improved emotional state, sense of belonging).

Some motivations to play, such as *achievement* and *immersion*, have been found to predict problematic engagement in MMORPGs [20,21,22,23,24], while this was not the case for the *recreation* motive [25]. Some studies have indicated that video games can improve socio-emotional well-being [26,27,28], and thus the excessive engagement in MMORPG can be conceptualized in certain circumstances as a maladaptive coping strategy to overcome anxiety and negative affect [29]. Experienced benefits could play the role of negative reinforcing factors and can be conceptualized as expected internal positive reward and may in some cases be linked to problematic involvement in video gaming.

### 1.4. Self-Regulation and Engagement in Gaming

From a cognitive point of view, addiction is the result of a dual process model where the balance is lost between two systems, the “impulsive” and the “reflective”, in favor of the former [30]. All addictions involve a disruption of the reward system which is intrinsically linked to motivation and emotional systems [31], thus the self-regulation of these systems is believed to play a key role in addictive behaviors [32]. This is not only because addiction affects the evaluation of relevant stimuli, but it is also suggested to influence automatic and controlled aspects of self-regulation [33]. Self-regulation is indeed an umbrella construct that refers to either automatic (or reactive/unconscious) and controlled (or deliberative/voluntary) processes [34,35,36].

Although existing studies highlight a consistent association between low self-regulation (reflected by high impulsivity and/or sensation seeking levels) and problematic engagement in online games, they often provide limited comprehension of the psychological mechanisms involved, as they have been conducted too often without considering the multifaceted nature of impulsivity [37] and decision-making [38].

#### 1.4.1. Automatic Aspects

Automatic aspects of self-regulation correspond to the reaction of motivational systems in response to a relevant stimulus and have to be related to the various processes (e.g., motor, perceptual) involved in approach behaviors and reinforcement seeking [36]. Automatic aspects of self-regulation have often been related to a high level of sensation seeking [36], defined as a proneness to look for novelty, complexity, and sensory stimulation with openness to new experiences [37]. A high level of sensation seeking was often yet non consistently found to be linked to addictive disorders [38]. Studies having investigated sensation seeking in GD are scarce, and its participation is still debated [39].

#### 1.4.2. Controlled Aspects

Controlled aspects of self-regulation depend on the efficacy of executive processes (e.g., inhibitory control) whereby an individual influences upon behaviors, thoughts, and emotions [34,36]. Since addictive behaviors are often described as impulse control disorders or at least as a deficit of the inhibitory control [40]. Through the use of different tools to assess its aspect, it appears that MMORPGs at risk of developing GD showed greater impulsivity, both in the UPPS [21] and via the BIS-11 [41].

### 1.5. Present Study

An online study was designed a decade ago to integrate various self-regulation aspects (automatic and controlled) whose impairment is a hallmark of addictive behaviors [42], and some specific determinants of gaming practice (motives to play and benefits from gaming), in order to identify psychological risk factors for and protective factors from problematic engagement in gaming in a large and representative sample of French adult gamers.

The first hypothesis (H1) is that there are some motives and benefits associated with gaming that can play the role of reinforcing factors in dysfunctional engagement, while some others can be protective from it.

The second hypothesis (H2) is that some particular impaired self-regulation aspects (impulsivity and sensation seeking components) can be predictors of dysfunctional engagement gaming.

These hypotheses were part of the initial protocol of the survey in 2008, and results have not yet been published even though the study ended in 2010.

We decided to publish these findings now, in consideration of the recently recognized diagnosis of GD in the ICD-11 [5]. Indeed, it resulted in an increased attention towards GD onset, clinical course, and treatment options. Clarifying the psychological underpinnings of its initiation and maintenance may be useful for clinical practice as it should help to refine the psychotherapeutic levers and goals [7,43]. Another current pressing issue since GD’s inclusion is to avoid over-pathologizing gaming activities in clinical practice. Data supporting the distinction between passion and addiction, will better inform clinical decision-making (i.e., to treat or not).

## 2. Materials and Methods

### 2.1. Participants and Procedure

French volunteer WoW (WoW) gamers 18 years of age and older were included in the study through an anonymous (no identifying data collected) recruitment in the game (private forums of gamers’ groups called “guilds” in WoW) between May 2009 and March 2010. No limit for time spent gaming was set for participation in the study.

A study teaser with a link to our online study called “Add MMORe”, was posted through an active personal gamer’s account into 234 MMORPGs’ guild forums. Upon accepting the invitation to participate, gamers could connect to the study website to sign the electronic consent after getting wild information on the procedure and the ethical cautions. Subjects received no compensation for their participation in the study. This study has received the approval of the Ethical Committee of Besancon University Hospital (authorization delivered by the General Health Administration: DGS2007-0382) and was declared to The National Commission of Informatics and Liberty CNIL on 25 July 2007. Study design and descriptive results (socio demographic data, screening for Internet Addiction and for Internet Gaming Disorder and reported associated consequences) of the whole sample (*n* = 448) have previously been published [20].

For the present dataset, 313 participants from the overall sample (*n* = 448) answered all the items considered to test the two hypotheses in the present work. Inclusion criteria were to be French, be a WoW gamer, be 18 or older, and be able to consent to participate. Participants which were minors or were not resident in French territories were excluded from the study. We did not apply any time limit of gaming in the recruitment procedure.

### 2.2. Measures

#### 2.2.1. Time Dedicated to MMORPGs

Time spent in online gaming was explored through self-reported hours per week played in the game WoW.

#### 2.2.2. Dysfunctional Engagement in MMORPGs, DSM IV-TR for Substance Dependence Adapted Scale (DAS)

Since the present study took place before IGD criteria introduced in DSM-5 (2013) and before GD criteria introduced in ICD-11 (2018), an adapted scale from DSM-IV-TR dependence criteria, the gold standard at that time for addictive disorders, was used.

DAS used in this study have been published in a previous paper as a good discriminating screening tool for addiction to MMORPGs [20]. It consisted of seven items (adapted from substance dependence DSM IV-TR criteria) related to online game use over the previous period of 12-months and answered «yes» or «no». For detailed information on DAS, see [20].

For the present paper, no cut-off for DAS was applied, and we considered engagement in WoW from a dimensional perspective, using the total score of DAS to assess severity, based on the number of items that were answered “yes” by the participants. The internal consistency in our sample, as measured by Cronbach’s alpha, was acceptable (α = 0.63). For regression analysis, items were assessed independently as dichotomous outcomes. They were referred to by their meaning as follows: “lack of time control” (LTC) (more than wanted), “lack of control on gaming activity” (LGC), “high involvement in gaming” (HI), “negative consequences” (NC), “gaming continued despite harms” (DH), “habituation” (Hab.) (need of more and more time gaming), and “withdrawal” (IR) (irritability, agitation).

#### 2.2.3. Expected Rewards

##### External Motives

We used a seven choice item developed for the study, with dichotomous response (yes/no), adapted from Yee’s motivations questionnaire [18] which has been fulfilled by participants to answer the question “What are you seeking in this online videogame?” Possible choices were “discovery or game environment exploration” (Discovery), “challenge” (Competition), “a role to play in an alternative world” (Role play), “interaction with other gamers” (Social), “having a powerful character” (Advancement) and “to escape from your identity or real-life” (Escapism). To Yee’s motivations, we added “recreation”, a motive we thought judicious as the most important motive to engage in a leisure activity as online gaming, that has been used by Demetrovics et al. [19].

##### Internal Motives

Internal motives are represented by the psychological benefits perceived or expected by participants in online games. Choices proposed were not directly mediated by game skills, and resulted from the feelings usually reported in relation to playing to MMORPGs in our clinical experience treating excessive gamers. Participants were then asked “What are the sensations online gaming provides you?”, and seven choices were proposed: “wellbeing”, “solace”, “enjoyment”, “less anxiety state”, “greater personal satisfaction”, “sense of power” and “sense of belonging to a group”. A dichotomous (yes/no) response was possible.

#### 2.2.4. Zuckerman Sensation Seeking Scale (ZSSS)

We used the French validated version [44] of the ZSSS [45,46], which is a 40-item scale with four subscales: Thrill and Adventure Seeking (TAS) desire to engage in sports or activities involving speed and danger (e.g., I often wish I could be a mountain climber), Experience Seeking (ES) desire for experience through the mind and senses, travel, and a non-conforming lifestyle (e.g., People should dress in individual ways even if the effects are sometimes strange), Disinhibition (Dis) desire for social and sexual disinhibition (e.g., I like wild “uninhibited” parties), and Boredom Susceptibility (BS) aversion to repetition, routine, and dull people (e.g., I can’t stand watching a movie that I’ve seen before). The internal consistency in our sample of the four subscales as well as for the total score were higher or equal than 0.5 (α_TAS_ = 0.79; α_ES_ = 0.55; α_DIS_ = 0.67; α_BS_ = 0.50; α_tot_ = 0.79).

#### 2.2.5. Barratt Impulsiveness Scale (BIS-10)

We used the 34 items using 4-point ratings (1 = never/rarely, 2 = occasionally, 3 = often, 4 = almost always/always) The French validated version [47] of the scale [48] was used to determine impulsivity levels. Three subscales correspond to motor impulsivity (MI) assesses tendency to act on the spur of the moment and consistency of lifestyle (e.g., I am restless at the theater or lectures), Attentional impulsivity (AI) assesses task-focus, intrusive thoughts, and racing thoughts (e.g., I buy things on impulse), and Non-planning impulsivity (NP) assesses careful thinking and planning and enjoyment of challenging mental tasks (e.g., I plan tasks carefully). We considered the global and the three subscales scores. The internal consistency in our sample of the three subscales as well as for the total score were higher or equal to 0.64 (α_MI_ = 0.69; α_AI_ = 0.70; α_NP_ = 0.64; α_tot_ = 0.80).

### 2.3. Statistical Analysis

Two-tailed point-biserial correlations or Pearson correlations were used to evaluate relationships between self-reported symptoms of dysfunctional gaming (according to DAS) or DAS severity and other variables of the study. The point-biserial correlation was used for evaluation when at least one variable was dichotomous. Subjects with missing data were excluded.

To assess the relationship between each predictor or risk factor and the dysfunctional involvement in MMORPGs, a logistic regression was performed. Each item of the DAS was entered as the dependent variable. Twenty-four independent predictors were entered in the model: age, gender, time spent gaming, motives to play online and benefits from gaming [24], impulsivity facets measured with the BIS-10 [49] and sensation-seeking facets measured by the ZSSS [50]. This analytical strategy was chosen to identify which specific DSM symptoms are influenced by the different predictors. The odds ratio allows for the assessment of the magnitude of the association between the variables and the covariates, taking into account the other variables in the model.

Analyses were performed with R version 4.1.2 [51] on R-Studio version 2021.09.2.382 [52].

## 3. Results

Participants in this sample (*n* = 313) were predominantly male (82.1%) and aged between 18 and 54 years old (M = 26.53, SD = 6.88). They played this online game an average of 29.45 h per week (SD = 17.65, range 5–100). They mainly reported being motivated to play the game by Recreation (78%), Social (77.6%), Competition (69%) and Discovery (66.5%), whereas other external motives such as Role play (31%), Advancement (25.2%), and Escapism (3.2%) were reported at lesser rates.

Participants also self-reported benefits from gaming, mainly enjoyment (83.1%), greater personal satisfaction (48.9%), sense of group belonging (40.9%), and wellbeing (36.4%). Online gaming was reported to reduce anxiety in 21.4%, to provide solace in 11.8%, and a sense of power in 8.6%.

The main findings from correlation analyses can be summarized at three distinct levels: demographic variables (age and gender), gaming variables (time spent, motives and benefits) and self-regulation psychometric variables. The correlations are reported in Table 1.

The logistic regression computed allowed us to highlight some predictors of dysfunctional engagement in MMORPGs (Table 2). Concerning demographic variables, the results presented a stronger tendency for females [OR = 2.39 *, IC = 1.20–4.85] and for older participants [OR = 1.08 ***, IC = 1.04–1.13] to experience lack of time control. Being a female also predicted a bigger risk of experiencing habituation symptoms [OR = 15.62 *, IC = 1.39–206.84], and older participants expressed a higher risk of experiencing negative consequences [OR = 1.07 *, IC = 1.00–1.14]. In addition, as expected, the highest t number of declared hours per week dedicated to gaming, the highest involvement in gaming [OR = 1.03 ***, IC = 1.02–1.05], continued playing despite harms [OR = 1.02 **, IC = 1.01–1.04] and withdrawal symptoms predicted [OR = 1.03 *, IC = 1.01–1.05] GD.

Advancement predicted lack of control in gaming activity [OR = 2.00 *, IC = 1.04–3.84], while competition predicted higher involvement in gaming activity [OR = 2.21 *, IC = 1.07–4.79] and continued playing despite harms [OR = 2.16 *, IC = 1.05–4.63]. Discovery motive, predicted habituation symptoms [OR = 8.10 *, IC = 1.29–81.79].

Moreover, some psychological benefits from gaming (internal motives) were associated with dysfunctional engagement in gaming; high involvement in gaming was predicted by the sense of power [OR = 3.14 *, IC = 1.15–8.95] and the greater personal satisfaction [OR = 2.12 *, IC = 1.15–3.95]. The existence of a motivation to game driven by the reduction of anxiety predicted negative consequences [OR = 2.49 *, IC = 1.03–5.99] along with withdrawal symptoms [OR = 3.40 **, IC = 1.46–7.97]. Lastly, the internal motive of the seeking of wellbeing predicted withdrawal symptoms [OR = 2.38 *, IC = 1.10–5.22].

Regarding the automatic aspects of self-regulation, thrill and adventure seeking predicted habituation [OR = 2.02 *, IC = 1.25–3.78], whereas, disinhibition predicted continued playing despite harms [OR = 1.16 *, IC = 1.01–1.35], and boredom susceptibility showed to be predictive of experiencing negative consequences from gaming [OR = 1.36 **, IC = 1.09–1.71]. However, experience seeking showed protective properties, with a tendency to negatively predict habituation symptoms [OR = 0.54 **, IC = 0.31–0.82], continued playing despite harms [OR = 0.75 ***, IC = 0.64–0.88] and lack of time control [OR = 0.87 *, IC = 0.76–1.00].

Finally, self-regulation controlled aspects that were also found to be predictors of dysfunctional engagement in gaming. Indeed, motor impulsiveness predicted continued playing despite harms [OR = 1.06 *, IC = 1.01–1.13] and habituation [OR = 1.25 **, IC = 1.07–1.50], whereas attentional impulsiveness predicted negative consequences [OR = 1.13 **, IC = 1.04–1.24], and the non-planning facet of impulsivity predicted habituation [OR = 1.15 *, IC = 1.01–1.34].

## 4. Discussion

The current work was a further investigation and integration in a subgroup (*n* = 313) of a representative and large sample of online video gamers recruited among the most popular MMORPG players (of WoW), having completed all the items of a set of psychological assessments administered during the overall survey (*n* = 448). The aim of the current dataset analysis, which was not previously published, was to unveil the interaction between controlled and automatic aspects of self-regulation, external and internal motives to game in one hand, and dysfunctional engagement in the game in another hand. As a reminder, this led us to formulate two different hypotheses, (H1) external motives to game and the benefits sought from gaming (internal motives) may play a protective and/or risk factor to dysfunctional engagement in video gaming, while (H2) aspects of impaired self-regulation may predict dysfunctional engagement in video gaming.

As hypothesized (H1), the study found some psychological factors related to online gaming (e.g., competition and advancement motives, reduced anxiety, solace, greater personal satisfaction, and sense of power) to be risk factors for several dysfunctional gaming items (e.g., dependence-like symptoms, continued playing despite harms, and high involvement) predicted dysfunctional gaming severity. Most of these predictive factors were found to be positively associated with pursuing gaming despite negative consequences. Competition also appears to be a risk factor for the development of the GD. This motivation has been previously identified to mediate between problematic gaming and general psychiatric distress [25]. As hypothesized, some of the external motives to play may indeed play the role of reinforcing factors, especially advancement and competition.

Besides that, new risk factors associated with GD and its severity were identified in the current study based on self-reports from participants. Thus, dysfunctional engagement in video gaming MMORPG can be understood as a maladaptive coping strategy to overcome anxiety and negative affect [29] in respondents.

Unlike the findings in the existing literature, we did not find that escapism was a risk factor for the development of GD, while we discovered that experiencing greater personal satisfaction constituted a risk factor for GD [24,25]. Indeed, while GD is often associated with escapism, i.e., relief from dissatisfaction, here we found an association with the desire for a greater personal satisfaction. These findings align with those observed in older internet users (aged above 60 years old) regarding the correlation between life dissatisfaction and internet addiction, and dysfunctional engagement online being an intent to improve well-being and life satisfaction for them [28]. The need for greater personal satisfaction could as well be related to aspects of the narcissistic personality already mentioned in the literature as being predictive of GD. Moreover, narcissistic personality traits and the propensity to be more aggressive [53] could be associated with the competition motive that can be defined as the will to play in order to defeat others and a way to get personal satisfaction at the expense of others. These aspects intersect with another psychological factor identified here as a risk factor for developing GD, the sense of power. However, it is important to make the distinction between advancement and the sense of power. Advancement is defined as “a facet of the higher-order dimension of achievement: the desire to become powerful and progress quickly in the game”, and that is translated here by the desire to have a powerful character [24]. Thus, gaining power is an essential component of success over other players and by consequence is an aspect of the competition [21,25], whereas the sense of power translates a positive feeling induced by these achievements and refers to an internal reward, which in this context is closer to the fact of satisfying a narcissistic dimension [53]. In addition, these reasons to play could be related to the concept of positive and negative reinforcement. More precisely, escapism could be interpreted as a negative reinforcement, (i.e., avoid dissatisfaction) and the desire for greater personal satisfaction as a positive reinforcement. This distinction seems to be crucial, as with other addictive disorders. Indeed, in alcohol use disorder, positive reinforcement is related to alcohol consumption, whereas negative reinforcement is related to alcohol dependence [54]. Along those lines, differences observed could be explained by clinical features in the sample studied. In our study, and in contrast with others, we did not identify socialization motives to be a risk factor [24,25]. Social motives are based on the desire to make new friends. On the other hand, GD is described to induce a social problem in real life which may increase this motivation by compensation [23,55]. Socialization could thus be a protective factor and the video game can create strong social bonds if players continue their friendships outside of online games, which improves socio-emotional well-being [28,29]. This positive aspect seems to be particularly true for players with significant motive to gain social capital and play as part of a team [26,27]. The socialization motive alone cannot help to define the risk to develop a dysfunctional gaming problem. In contrast, how the social bonds are created and maintained can better inform about the risks of developing GD.

This study also found some protective psychological factors (e.g., recreation, enjoyment and experience seeking) with a predominant protective property from continued gaming despite harms. The term Recreation traduced “*motivation for playing video game for fun*” [25] and by consequence intersect the enjoyment item. Thus, gaming for enjoyment and recreation are protective factors against GD. This reminds us that the video game is above all a form of entertainment and that many gamers with an increased involvement in video games are not necessarily affected by GD [2,56,57]. This highlights the need to distinguish between passionate and disordered gamers [2,22].

These results could identify functional gamers and dysfunctional or lower-risk gamers based on a simple discourse analysis. Thus, when gamers report being motivated to play for fun and experience seeking, they are likely functional., whereas some motivations provide arguments for dysfunctional activity such as certain positive reinforcers (competition, enhancement of self-satisfaction, advancement or sense of power) or negative reinforcers (decreased anxiety and increased wellbeing). Furthermore, very specific associations were found between negative reinforcing factors (e.g., solace and reduced anxiety sought benefits from gaming) and loss of control with regard to gaming time. Despite the fact that these results are not significant in the logistic regression, they show the biggest effect size regarding the lack of time control. This suggests that gaming more than one wants occurs when the gamer is seeking to alleviate negative emotional states. These findings are very interesting because they highlight the heterogeneous nature of dysfunctional gaming. Some gamers could have developed problematic gaming as a dysfunctional coping strategy against anxiety symptoms, while others developed it to improve their self-esteem. The existence of different dysfunctional gamers’ profile is in line with recent cluster approach findings [2]. At the time, only external motives to play) have been investigated as predictors of problematic gaming specific to MMORPGs. The present work provides additional motivational predicting factors for GD, those related to internal motives, including anxiety relief and the seeking of solace) that could be targeted by specific psychotherapeutic approaches to treat dysfunctional gaming regardless of the game genre. While the existence of psychological symptoms (depression, anxiety) related to GD is known, the report of gamers using video gaming to regulate anxiety and seek solace is new. We hereby present, in the appendix section the items we developed to assess internal motives, which could guide clinical practice.

Our hypothesis H2 has been verified, with some self-regulation aspects being predictors for dysfunctional involvement in MMORPGs. Regarding automatic aspects, we observed that susceptibility to boredom was associated with negative consequences from gaming and withdrawal (irritability) symptoms. Disinhibition predicted negative consequences and continued gaming despite harms, the latter being significant both in correlation and in logistic regression analysis. The thrill and adventure seeking item was correlated to high involvement in gaming but predicted only habituation symptoms. Interestingly, experience-seeking showed protective properties from all criteria of the DAS except for high involvement in gaming, with continuing gaming despite harms being the most significant. As a reminder, the search for experience has translated into a search for novelty and an unconventional lifestyle. In contrast, Disordered gamers are known to show a lower level of extraversion and openness to new experiences [58]. It is thus not surprising that experience-seeking appears to be a protective factor from GD. While individuals with GD have a lower propensity to be open to new experiences (i.e., have shown lower experience-seeking), we observe on the other hand a higher level of boredom susceptibility, disinhibition, and also thrill and adventure seeking. To better interpret these data, it seems important to focus on the definition of each sensation- seeking component. Indeed, boredom susceptibility translates to an aversion to routine activities, while thrill and adventure seeking translates to the attraction to thrills such as risky sports [39], and the disinhibition aspect refers to the adoption of an extraverted attitude, a social disinhibition a variety of sexual needs and the use of psychoactive substances [59]. It is with regard to the disinhibition component that the literature has shown the most contradictory results. While MMORPG gamers have been described as shy and socially insecure, a psychological aspect congruent with a weaker disinhibition observed in the literature on GD, other studies have found a stronger disinhibition [60], which is in line with our findings. However, the populations in these studies are composed of individuals with either an Internet addiction, a personality disorder, or both, which could be confusing. In addition, we have previously found that experience seeking was identified as a protective factor from GD; in other words, individuals suffering from GD expressed a lower tendency towards experience seeking. Despite this lesser tendency to seek novelty and embrace unconventional living, they showed a stronger search for thrills and adventure. These aspects appear very interesting because they allow for the questioning of the role and the utility of virtual life. Indeed, virtual universes could appear as a good compromise to satisfy thrill, and adventure seeking and a way of fighting boredom without exposing an individual to a feared unknown social environment. Notably, the sensation seeking provides a coping mechanism to overcome boredom [61]. This is concordant with our clinical observations. Patients seeking treatment for online video gaming addiction often declare negative consequences being the triggering factor for seeking help, and they often report important intolerance to boredom and a very negative inner experience of it [62,63].

Nevertheless, it cannot be excluded that other sensation seeking facets could be at-risk factors for dysfunctional gaming. The Zuckerman scale has in fact since the end of the present survey been criticized as perhaps not being tailored to measure the kind of stimulations and rewards sought in problematic internet use [36]. This could be due to the scale’s nature, as it was created in the 1960s and was not adapted to the sensations sought in gaming. Then, external and internal motives to play could be good candidates for developing questionnaires (sensation seeking and reward sensitivity) adapted to MMORPG gamers. These tools could be coupled with laboratory tasks to further clarify the automatic aspects of self-regulation [36].

Regarding the controlled aspects of self-regulation, impulsivity (BIS-10) was linked to dysfunctional and severe involvement in gaming. Since pretty much all facets of impulsivity have correlated to each criterion of the DAS, we will focus on the results of the logistic regression, which takes into account the other variables in the model. These links between impulsivity assessed by the BIS-10 and the severity of dysfunctional gaming have been previously reported [9,64,65]. Regarding attentional impulsivity, which reflects difficulty maintaining attention to relevant details [66], it predicted significantly negative consequences. Thus, the individuals scoring for GD exhibited attentional impulsivity that impaired their ability to focus on relevant information and become aware of current and future difficulties. Motor impulsivity related to the inhibition of pre-potent behavior could be translated into the tendency to adopt impulsive behavior or to act without thinking [48,66]. Motor impulsivity predicted pursuing gaming despite harms and habituation symptoms, and the non-planning facet of impulsivity predicted habituation symptoms. These were two impulsivity components previously reported as associated with GD [9]. The non-planning impulsivity translates to the lack of foresight and a tendency not to plan or consider consequences before starting something [48]. Motor impulsivity could be explained by the resulting very fast motor response to the gaming urge, challenging inhibition capacities. On the other hand, the non-planning facet of impulsivity predicted habituation symptoms, suggesting that when the gaming activity is unplanned, the gamer might rather cede to the envy of gaming, resulting in repeated exposition. It could then become recurrent and result in the need to game more and more. In other words, gaming pre-potent response inhibition in this case could occur too late once the motor response to the gaming need has been satisfied. The urge to game as a coping emotional strategy (driven by internal motives) could contribute to the gaming control failure and dysfunctional gaming, especially in high emotional contexts. This is quite consistent with the idea that repetition of a rewarding form of behavior could lead to its automatization and the concomitant resulting loss of control in GD. The impulsive system mediates fast, automatic, unconscious, and recurrent behaviors [67]. It is important to weigh this information, particularly on attention and the tendency to act quickly, having in mind that video games are well known to improve certain cognitive functions including attention, spatial cognition and response time [68,69,70]. These results are consistent with those of previous studies that used a different measure of impulsivity but found a higher proportion of dysfunctional gamblers to act recklessly, particularly in response to negative affect [21], and more specifically to show both abnormalities in reward systems and inhibitory control [65].

This should be replicated and further assessed by objective cognitive tasks as proposed in the study of Yan et al. [65], and adding the specific internal and external motives that lead people to game and to adopt dysfunctional gaming. Some clinical implications for cognitive and behavioral therapy applied to online gaming disorders could result from this. Cognitive focus could consist in identifying high emotional contexts and emotional coping strategies to design psychotherapy sessions targeting urgency reduction. A behavioral focus could consist in progressively training patients to delay the gaming behavior when the desire to game appears. Finally, specific benefits for the problematic gamer should be paid attention to in psychotherapy, as they offer understanding about psychological mechanisms related to problematic gaming onset and relapse, such as feelings of loneliness, un-relatedness, low self-esteem or anxiousness [16,56,71,72]. Treating those risk-factors would guarantee clinical improvement and relapse prevention. These personalized psychotherapeutic approaches are needed for GD and more broadly for Problematic Internet Use (PIU). As previously stated in the recent literature [56,71]. Lastly, while the scientific community as a whole agrees that time spent and frequency of gaming cannot be used as a diagnostic criterion, many have suggested that they should be considered as a monitoring tool for gamers [72]. In the present work, we observed that our sample showed a positive correlation between the number of hours per week dedicated to gaming and the involvement in gaming, and the tendency to continue playing despite harms and withdrawal symptoms. More precisely, the number of hours devoted weekly to WoW moderately correlated to gaming severity and all dysfunctional gaming involvement items except for the two items related to gaming control. To summarize our findings and to highlight the relevant ones for clinical practice, we provide, in Appendix A, all protective and risk factors associated with GD from internal and external motives to self-regulation characteristics. We also provide an Appendix B, which lists the items exploring internal and external motives, as we used them in our study in French and in the English translation to guide clinical interviews aimed at differentiating between passionate gaming and dysfunctional gaming patterns.

Limitations of the present work consist in the self-selected sample, the self-reported nature of the administered questionnaires, and the binary nature of motives and benefits items (yes/no). Another limitation is the timing of the data collection (2009–2010). However, we found that our sample still seems representative of the MMORPG population. Indeed, while the video game industry has undergone a great change over the past decade, we observe that these changes affect MMORPGs little or not at all. World of Warcraft remains very popular [73] and is not subject to changes such as monetization or the access through smartphones [73,74]. In addition, our socio-demographic data confirmed that our sample is representative with results in line with previous studies. Our sample, even if it consisted of a large number of young men [14,75], presented, however, an overall similar amount of time spent gaming online, as in previous studies [76]. In addition, certain demographics were already identified in the literature as being associated with dysfunctional gaming behavior [71,77]. Women showed a higher risk for experiencing habituation symptoms [77], while older gamers expressed a higher risk of experiencing negative consequences [71]. Females and older gamers both showed a higher tendency to experience lack of time control and to continue gaming despite observed harms related to their gaming activity. Younger players tended to need more and more gaming activity and to experience irritability and agitation when they could not game.

Motives to game and motor impulsivity could be predictors of excessive online gaming and addiction maintaining factors, probably differing from a videogame category to another (war-games, role playing games, strategy games, etc.), and justifying the assessment of each subgroup of online gamers.

Finally, further studies could benefit from presenting findings on rewards sought by MMORPG players and self-regulation capacities to further explore these aspects with experimental designs (laboratory tasks).

## 5. Conclusions

Gaming is a very popular leisure activity; however, gaming disorder, recently acknowledged by the WHO as an addictive mental health condition, can affect a minority of users. A current pressing need in clinical practice is to have landmarks on the gaming patterns which are functional and those which are at risk for GD.

The current study adds knowledge on the way to distinguish the passionate from the addictive pattern of gaming. Beyond time spent gaming, further investigation is needed in terms of clinical interviewing, of underlying internal and external motives, and self-regulation aspects.

We confirmed that motivations related to some external rewards (such as competition and advancement) and some internal rewards (such as solace, reduced anxiety, greater personal satisfaction and sense of power) should be identified as at-risk factors for GD and should be addressed in treatment, by adapting cognitive-behavioral interventions, for example. Furthermore, we showed that considering a symptomatic approach to dysfunctional gaming (DAS items) can be helpful to unveil specificities related to different age groups and gender in psychotherapy of GD.

Protective factors we identified can serve as a blueprint for qualifying functional gaming and for counselling parents on healthy technology use.

## Figures and Tables

**Table 1:** Correlations between the symptoms of engagement in gaming according to DAS and demographics and psychological variables (N = 313).

Variables	DAS-LTC ^a^	DAS-LGC ^a^	DAS-HI ^a^	DAS-NC ^a^	DAS-DH ^a^	DAS-Hab ^a^	DAS-IR ^a^	DAS Severity
Demographics								
Age	0.11	−0.05	−0.05	−0.04	−0.17 **	−0.13 *	−0.13 *	−0.09
Gender ^a^ (ref. = male)	0.09	−0.05	0.01	−0.04	−0.11 *	0.00	0.05	−0.03
Time spent (hours/week)	0.08	0.05	0.29 ***	0.18 **	0.27 ***	0.23 ***	0.26 ***	0.31 ***
External motives								
Discovery	−0.02	−0.10	0.00	−0.05	−0.10	0.08	−0.04	−0.07
Recreation	0.00	−0.09	−0.09	−0.15 *	−0.21 ***	−0.08	−0.09	−0.16 **
Competition	0.03	0.07	0.21 ***	0.11	0.18 **	0.13 *	0.12 *	0.21 ***
Role-play	−0.02	−0.05	0.08	0.03	0.09	0.14 *	0.12 *	0.07
Social	−0.05	−0.05	0.05	−0.05	−0.07	−0.04	−0.02	−0.05
Advancement	0.00	0.19 ***	0.20 ***	0.13 *	0.14 *	0.09	0.14 *	0.23 ***
Escapism	0.00	−0.07	−0.12 *	−0.07	−0.08	0.12 *	−0.02	−0.08
Internal motives								
Wellbeing	0.00	−0.04	0.00	0.02	0.01	0.08	0.14 *	0.04
Solace	0.11 *	0.03	0.09	0.00	0.12 *	0.04	0.13 *	0.14 *
Enjoyment	−0.06	−0.04	0.00	−0.09	−0.16 **	−0.08	−0.06	−0.12 *
Reduced anxiety	0.12 *	0.08	0.05	0.11	0.11	0.18 **	0.21 ***	0.22 ***
Greater personal satisfaction	0.06	0.11 *	0.24 ***	0.15 **	0.14 *	0.10	0.11	0.23 ***
Sense of power	0.01	0.10	0.22 ***	0.08	0.17 **	0.18 **	0.13 *	0.22 ***
Sense of group belonging	−0.01	−0.05	0.06	0.07	0.08	−0.06	−0.01	0.04
Self-regulation (automatic aspects)								
ZSSS—Total score	0.02	0.01	0.10	0.11 *	0.00	0.08	0.01	0.08
ZSSS—TAS	0.03	0.00	0.12 *	0.01	0.00	0.12 *	−0.01	0.07
ZSSS—ES	−0.04	−0.10	0.07	−0.02	−0.18 **	−0.06	−0.08	−0.10
ZSSS—Dis	0.04	0.04	0.06	0.11 *	0.13 *	0.05	0.02	0.12 *
ZSSS—BS	0.01	0.09	0.02	0.19 ***	0.04	0.07	0.11 *	0.12 *
Self-regulation (controlled aspects)								
BIS—Total score	0.17 **	0.16 **	0.14 *	0.24 ***	0.23 ***	0.24 ***	0.16 **	0.32 ***
BIS10—MI	0.15 **	0.15 **	0.15 **	0.20 ***	0.23 ***	0.25 ***	0.20 ***	0.33 ***
BIS10—AI	0.16 **	0.15 **	0.07	0.26 ***	0.16 **	0.19 **	0.16 **	0.28 ***
BIS10—NP	0.08	0.08	0.11 *	0.13 *	0.16 **	0.14 *	0.02	0.17 **

*** *p* < 0.001 ** *p* < 0.01 * *p* < 0.05; ^a^ dichotomous variables; DAS severity (ln items + 1); LTC = lack of time control, LGC = lack of control on gaming activity, HI = high involvement in gaming, NC = negative consequences, DH = gaming continued despite harms, Hab = habituation, IR = withdrawal symptoms, ZSSS = Zuckerman sensation seeking scale, ZSSS-TAS = thrill and adventure seeking, ZSSS-ES = experience seeking, ZSSS-Dis = disinhibition, ZSSS-BS = boredom susceptibility, BIS10 = Barratt Impulsiveness Scale, BIS10-MI = motor Impulsivity, BIS10-AI = attentional impulsivity, BIS10-NP = non-planning.

**Table 2:** Logistic regression results (N = 313).

	DAS-LTC ^a^	DAS-LGC ^a^	DAS-HI ^a^	DAS-NC ^a^	DAS-DH ^a^	DAS-Hab ^a^	DAS-IR ^a^
	OR [95% CI]	OR [95% CI]	OR [95% CI]	OR [95% CI]	OR [95% CI]	OR [95% CI]	OR [95% CI]
age	1.08 *** [1.04;1.13]	1.01 [0.97;1.06]	1.03 [0.98;1.08]	1.07 * [1.00;1.14]	1.01 [0.96;1.06]	0.97 [0.83;1.11]	1.00 [0.93;1.07]
Gender ^a^	2.39 * [1.20;4.85]	1.10 [0.48;2.41]	1.83 [0.81;4.08]	1.58 [0.49;4.62]	0.74 [0.29;1.76]	15.62 * [1.39;206.84]	2.83 [0.98;8.06]
Hours/week in game	1.01 [0.99;1.02]	1.00 [0.98;1.01]	1.03 *** [1.02;1.05]	1.02 [1.00;1.04]	1.02 ** [1.01;1.04]	1.03 [1.00;1.07]	1.03 * [1.01;1.05]
Discovery	0.99 [0.57;1.72]	0.81 [0.45;1.50]	0.85 [0.45;1.63]	0.79 [0.34;1.91]	0.70 [0.37;1.31]	8.10 * [1.29;81.79]	0.78 [0.34;1.82]
Recreation	1.05 [0.54;2.04]	0.85 [0.42;1.76]	1.14 [0.54;2.45]	0.66 [0.26;1.72]	0.56 [0.28;1.15]	0.76 [0.13;4.96]	0.74 [0.29;1.95]
Competition	1.57 [0.88;2.87]	1.22 [0.62;2.45]	2.21 * [1.07;4.79]	1.59 [0.59;4.60]	2.16 * [1.05;4.63]	9.24 [0.85;314.07]	2.42 [0.90;7.21]
Role Play	0.87 [0.49;1.54]	0.67 [0.34;1.27]	1.21 [0.62;2.34]	1.13 [0.47;2.67]	1.45 [0.75;2.80]	2.22 [0.49;11.18]	1.35 [0.58;3.11]
Social	0.57 [0.30;1.07]	0.69 [0.35;1.40]	1.20 [0.57;2.61]	0.65 [0.26;1.72]	0.84 [0.40;1.79]	1.12 [0.15;11.23]	0.99 [0.38;2.73]
Advancement	0.75 [0.39;1.40]	2.00 * [1.04;3.84]	1.56 [0.78;3.09]	1.71 [0.71;4.10]	0.86 [0.42;1.73]	0.97 [0.19;5.10]	1.11 [0.46;2.61]
Escapism	0.80 [0.19;3.29]	0.32 [0.02;2.00]	--	--	0.26 [0.01;1.70]	--	0.41 [0.02;3.33]
Well-Being	0.95 [0.55;1.62]	0.80 [0.43;1.46]	0.63 [0.33;1.17]	1.18 [0.52;2.65]	0.89 [0.47;1.65]	4.28 [0.97;23.81]	2.38 * [1.10;5.22]
Solace	1.80 [0.77;4.34]	1.04 [0.41;2.57]	1.47 [0.57;3.73]	0.40 [0.10;1.35]	1.96 [0.78;4.94]	0.24 [0.02;1.83]	0.82 [0.26;2.36]
Enjoyment	0.94 [0.46;1.92]	1.19 [0.55;2.69]	0.91 [0.40;2.12]	0.84 [0.31;2.41]	0.68 [0.31;1.49]	0.19 [0.02;1.46]	0.99 [0.37;2.85]
Diminution of Anxiety	1.89 [1.00;3.65]	1.49 [0.75;2.93]	1.16 [0.56;2.37]	2.49 * [1.03;5.99]	1.90 [0.93;3.89]	4.61 [0.94;25.61]	3.40 ** [1.46;7.97]
Personal Satisfaction increase	1.16 [0.68;2.00]	1.39 [0.76;2.55]	2.12 * [1.15;3.95]	2.06 [0.90;4.85]	0.94 [0.49;1.78]	2.32 [0.42;15.11]	1.29 [0.57;2.94]
Sense of power	1.06 [0.40;2.81]	1.07 [0.40;2.78]	3.14 * [1.15;8.95]	0.69 [0.18;2.29]	1.92 [0.67;5.60]	2.23 [0.34;14.33]	1.24 [0.37;3.86]
Sense of group belonging	0.97 [0.57;1.63]	0.85 [0.47;1.53]	1.40 [0.76;2.57]	2.09 [0.96;4.67]	1.46 [0.80;2.70]	0.50 [0.08;2.56]	1.03 [0.46;2.31]
ZSSS-TAS	1.11 [0.97;1.27]	1.03 [0.89;1.19]	1.16 [0.99;1.37]	1.05 [0.86;1.29]	1.01 [0.87;1.18]	2.02 * [1.25;3.78]	0.97 [0.79;1.19]
ZSSS-ES	0.87* [0.76;1.00]	0.89 [0.77;1.03]	1.14 [0.97;1.35]	0.91 [0.75;1.11]	0.75 *** [0.64;0.88]	0.54 ** [0.31;0.82]	0.84 [0.69;1.02]
ZSSS-Dis	1.03 [0.91;1.16]	0.99 [0.86;1.13]	0.93 [0.81;1.07]	0.97 [0.81;1.16]	1.16* [1.01;1.35]	0.82 [0.56;1.16]	0.99 [0.83;1.18]
ZSSS-BS	0.95 [0.82;1.10]	1.06 [0.90;1.25]	0.98 [0.82;1.16]	1.36 ** [1.09;1.71]	0.92 [0.77;1.09]	0.82 [0.50;1.33]	1.19 [0.95;1.49]
BIS10-MI	1.04 [1.00;1.10]	1.04 [0.99;1.09]	1.03 [0.97;1.09]	1.00 [0.93;1.08]	1.06 * [1.01;1.13]	1.25 ** [1.07;1.50]	1.06 [0.99;1.14]
BIS10-AI	1.05 [1.00;1.11]	1.03 [0.97;1.09]	0.98 [0.92;1.04]	1.13 ** [1.04;1.24]	0.99 [0.93;1.05]	0.98 [0.85;1.11]	1.03 [0.96;1.12]
BIS10-NP	1.02 [0.97;1.07]	0.99 [0.94;1.04]	1.05 [0.99;1.10]	1.00 [0.93;1.07]	1.03 [0.98;1.09]	1.15 * [1.01;1.34]	0.96 [0.89;1.03]

*** *p* < 0.001 ** *p* < 0.01 * *p* < 0.05; ^a^ dichotomous variables; LTC = lack of time control, LGC = lack of control on gaming activity, HI = high engagement in gaming, NC = negative consequences, DH = gaming continued despite harms, Hab = habituation, IR = withdrawal symptoms, ZSSS = Zuckerman sensation seeking scale, ZSSS-TAS = thrill and adventure seeking, ZSSS-ES = experience seeking, ZSSS-Dis = disinhibition, ZSSS-BS = boredom susceptibility, BIS10 = Barratt Impulsiveness Scale, BIS10-MI = motor impulsivity, BIS10-AI = attentional impulsivity, BIS10-NP = non-planning.

## Data Availability

Not applicable.

## Abbreviations

Add MMORe	Addiction and Massively Multiplayer Online Roleplaying games Research
AI	Attentional Impulsivity (BIS-10)
BIS-10	Barratt Impulsiveness Scale
BS	Boredom Susceptibility (ZSSS)
CNIL	National Commission of Informatics and Liberty (France)
DAS	Dependence Adapted Scale
DH	Gaming continued Despite Harms (DAS scale)
Dis	Disinhibition (ZSSS)
DSM-IV TR	Diagnostic and Statistical Manual of Mental Disorders, 4th ed., text rev.
DSM-5	Diagnostic and Statistical Manual of Mental Disorders, 5th Ed.
ES	Experience Seeking (ZSSS)
GD	Gaming Disorder
Hab	Habituation (DAS)
HI	High Involvement in gaming (DAS)
ICD-11	International Classification of Diseases 11th revision
IGD	Internet Gaming Disorder
IR	Withdrawal (Irritability) (DAS)
LGC	Lack of Control on Gaming activity (DAS)
LTC	Lack of Time Control (DAS)
MI	Motor Impulsivity (BIS-10)
MMORPGs	Massively Multiplayer Online Role-Playing Games
NC	Negative Consequences (DAS)
NP	Non-Planning Impulsivity (BIS-10)
PIU	Problematic Internet Use
SUD	Substance Use Disorders
TAS	Thrill and Adventure Seeking (ZSSS)
UPPS	Impulsive Behavior Scale (Urgency, Premeditation, Perseverance, Sensation Seeking)
WHO	World Health Organization
WoW	World of Warcraft
ZSSS	Zuckerman Sensation Seeking Scale

## Appendix A. Summary of Findings Providing Insights for Clinical Practice

**Table:**

**Variables**							**DAS Severity**
**PROTECTIVE FACTORS**							
External motives							
Recreation							−0.16 **
Internal motives							
Enjoyment							−0.12 *
**RISK FACTORS**							
Demographics							
Time spent gaming (hours/week)							0.31 ***
External motives							
Competition							0.21 ***
Advancement							0.23 ***
Internal motives							
Solace							0.14 *
Reduced anxiety							0.22 ***
Greater personal satisfaction							0.23 ***
Sense of power							0.22 ***
Self-regulation (automatic aspects)							
Disinhibition							0.12 *
Boredom susceptibility,							0.12 *
Self-regulation (controlled aspects)							
Barratt Impulsiveness Scale—Total score							0.32 ***
Motor impulsivity,							0.33 ***
Attentional impulsivity							0.28 ***
Non-planning							0.17 **

*** *p* < 0.001, ** *p* < 0.01, * *p* < 0.05; dichotomous variables.

## Appendix B

### Appendix B.1. External Motives

**French Version**:

Que recherchez-vous dans les MMORPG (Plusieurs réponses possibles)?

La découverte et l’exploration d’un univers de jeu

Un moment de détente

Un challenge ou un défi à relever

L’incarnation d’un rôle dans un monde alternatif

L’interaction avec d’autres joueurs

Avoir un personnage puissant

Fuir votre identité et/ou votre vie réelle

**English version**:

What are you seeking in MMORPGs? (Multiple responses are possible)

Discovery and exploration of the game environment

Recreation

A challenge

Embodying a role in an alternative world

Interaction with other gamers

Having a powerful game character

Escaping from your identity or real-life

### Appendix B.2. Internal Motives

**French version**:

Quelles sensations vous procure le jeu ? (plusieurs réponses possibles)

Bien être

Réconfort

Plaisir

Diminution de l’anxiété

Satisfaction personnelle

Sentiment de puissance

Sentiment d’appartenir à un groupe

**English version**:

What are the sensations gaming provides you? (Multiple responses are possible)

Wellbeing

Solace

Enjoyment

Reduced anxiety

Greater personal satisfaction

Sense of power

Sense of belonging to a group
